# Supplementary material for: Acute Mountain Sickness and the Risk of Subsequent Psychiatric Disorders—A Nationwide Cohort Study in Taiwan
Source: Int J Environ Res Public Health. 2023 Feb 6;20(4):2868. doi: 10.3390/ijerph20042868 (PMC9957283; doi:10.3390/ijerph20042868)
Supplement: Supplementary file 1 [file ijerph-20-02868-s001.zip › Table S1.pdf]

Table S1. Numbers of psychiatric disorders in the tracking of 16 years among the acute mountain sickness cohort and the control group.

**AMS**	**With (n = 127)**	**Without (n = 1270)**	**Log-Rank Test** *p*
**In the Tracking of x year(s)**	**Numbers of Psychiatric Disorders**		
**1**	21	11	<0.001
**2**	29	29	<0.001
**3**	31	48	<0.001
**4**	36	55	<0.001
**5**	39	71	<0.001
**6**	40	82	<0.001
**7**	41	94	<0.001
**8**	43	97	<0.001
**9**	44	103	<0.001
**10**	44	110	<0.001
**11**	46	114	<0.001
**12**	48	122	<0.001
**13**	49	130	<0.001
**14**	49	134	<0.001
**15**	49	139	<0.001
**16**	49	140	<0.001

There was a significant difference of cumulative survival between the two cohorts in the psychiatric disorders (log-rank test, $p < 0.001$). Abbreviations: AMS, Acute Mountain Sickness.
