# Supplementary material for: Acute Mountain Sickness and the Risk of Subsequent Psychiatric Disorders—A Nationwide Cohort Study in Taiwan
Source: Int J Environ Res Public Health. 2023 Feb 6;20(4):2868. doi: 10.3390/ijerph20042868 (PMC9957283; doi:10.3390/ijerph20042868)
Supplement: Supplementary file 1 [file ijerph-20-02868-s001.zip › Table S2.pdf]

Table S2. Sensitivity test for factors of the development of psychiatric disorders by the Fine & Gray's competing risk model among the acute mountain sickness cohort and the control group.

	**AMS**	**With**		**Without** *(Reference)*		***Competing risk in the model***			
**Sensitivity test**	**Psychiatric disorders**	**Events**	**Rate (per 10^5 PYs)**	**Events**	**Rate (per 10^5 PYs)**	**Adjusted sHR**	**95% CI**	**95% CI**	***p***
Overall	Overall	49	4,175.58	140	1,126.19	10.384	7.267	14.838	<0.001
	Anxiety	13	1,107.81	22	176.97	17.531	12.269	25.051	<0.001
	Depression	12	1,022.59	17	136.75	20.943	14.656	29.925	<0.001
	Bipolar	4	340.86	4	32.18	29.669	20.763	42.394	<0.001
	Sleep disorders	10	852.16	35	281.55	8.477	5.932	12.113	<0.001
	PTSD / ASD	2	170.43	1	8.04	59.337	41.526	84.789	<0.001
	Psychotic disorders	2	170.43	13	104.57	4.564	3.194	6.522	<0.001
	Schizophrenia	1	85.22	7	56.31	4.238	2.966	6.056	<0.001
	Other psychotic disorders	1	85.22	6	48.27	4.945	3.460	7.066	<0.001
	SRD	13	1,107.81	27	217.19	14.285	9.997	20.412	<0.001
	AUD	8	681.73	23	185.02	10.320	7.222	14.746	<0.001
	IDUD	5	426.08	4	32.18	37.086	25.954	52.993	<0.001
In the first year excluded	Overall	28	2,452.13	129	1,046.05	6.565	4.594	9.381	<0.001
	Anxiety	7	613.03	18	145.96	11.763	8.232	16.808	<0.001
	Depression	8	700.61	16	129.74	15.123	10.584	21.610	<0.001
	Bipolar	1	87.58	4	32.44	7.562	5.292	10.805	<0.001
	Sleep disorders	9	788.18	32	259.49	8.507	5.953	12.156	<0.001
	PTSD / ASD	1	87.58	1	8.11	30.247	21.167	43.220	<0.001
	Psychotic disorders	2	175.15	11	89.20	5.499	3.849	7.858	<0.001
	Schizophrenia	1	87.58	7	56.76	4.321	3.024	6.174	<0.001
	Other psychotic disorders	1	87.58	4	32.44	7.562	5.292	10.805	<0.001
	SRD	5	437.88	27	218.94	5.601	3.920	8.004	<0.001
	AUD	2	175.15	23	186.51	2.630	1.841	3.758	<0.001
	IDUD	3	262.73	4	32.44	22.685	15.876	32.415	<0.001
In the first 5 years excluded	Overall	10	1,317.55	69	630.71	5.851	4.094	8.360	<0.001
	Anxiety	4	527.02	7	63.98	23.068	16.144	32.963	<0.001
	Depression	3	395.27	10	91.41	12.111	8.475	17.305	<0.001
	Sleep disorders	3	395.27	21	191.95	5.767	4.036	8.241	<0.001
	SRD	2	263.51	10	91.41	8.074	5.650	11.537	<0.001
	AUD	2	263.51	10	91.41	8.074	5.650	11.537	<0.001

Abbreviations: AMS, Acute Mountain Sickness; Adjusted sHR, Adjusted sub-distribution Hazard ratio, Adjusted for the variables listed in Table 2 ; CI, confidence interval; PTSD/ASD, post-traumatic stress disorder/acute stress disorder; SRD, substance-related disorder; AUD, alcohol use disorder; IUD, illicit drug use disorder. Competing variable: all-cause mortality.
