# Supplementary material for: Acute Mountain Sickness and the Risk of Subsequent Psychiatric Disorders—A Nationwide Cohort Study in Taiwan
Source: Int J Environ Res Public Health. 2023 Feb 6;20(4):2868. doi: 10.3390/ijerph20042868 (PMC9957283; doi:10.3390/ijerph20042868)
Supplement: Supplementary file 1 [file ijerph-20-02868-s001.zip › Table S3.pdf]

Table S3. Years to the development of psychiatric disorders among the acute mountain sickness cohort and the control group.

AMS	Min	Median	Max	Mean ± SD
With	0.02	1.66	12.37	2.96 ± 3.56
Without	0.56	4.86	15.26	5.95 ± 4.23
Total	0.02	4.15	15.26	5.17 ± 4.26

Abbreviations: AMS, Acute Mountain Sickness; SD, standard deviation.
