# Supplementary material for: Acute Mountain Sickness and the Risk of Subsequent Psychiatric Disorders—A Nationwide Cohort Study in Taiwan
Source: Int J Environ Res Public Health. 2023 Feb 6;20(4):2868. doi: 10.3390/ijerph20042868 (PMC9957283; doi:10.3390/ijerph20042868)
Supplement: Supplementary file 1 [file ijerph-20-02868-s001.zip › Table S4.pdf]

Table S4. Sensitivity test for factors of the development of psychiatric disorders by the Fine & Gray's competing risk model among the acute mountain sickness cohort and the control group.

	AMS	With		Without *(Reference)*		*Competing risk in the model*			
**Sensitivity test**	**Psychiatric disorders**	**Events**	**Rate (per 10^5 PYs)**	**Events**	**Rate (per 10^5 PYs)**	**Adjusted sHR**	**95% CI**	**95% CI**	***P***
Overall	Overall	49	4,175.58	140	1,126.19	10.384	7.267	14.838	<0.001
	Single psychiatric diagnosis	21	1,789.54	63	506.78	9.890	6.921	14.131	<0.001
	Multiple psychiatric diagnoses	28	2,386.05	77	619.40	10.789	7.550	15.416	<0.001

Abbreviations: AMS, Acute Mountain Sickness; Adjusted sHR, Adjusted sub-distribution Hazard ratio, Adjusted for the variables listed in Table 2 ; CI, confidence interval; Competing variable: all-cause mortality.
